# Dental Materia Medica

**Published:** 1866-02

**Authors:** Geo. Watt


### DENTAL REGISTER.

VOL. XX.] FEBRUARY, 1866. [No. 2.

### Original Communications.

### DENTAL MATERIA MEDICA.

By Geo. Watt.

#### CALVERT'S CARBOLIC ACID.

THIS compound is but little known in the profession, and has, consequently, not received the attention to which its merits entitle it. I am not acquainted with its chemical formula. It being one of the many compounds resulting from the destructive distillation of organic matter, its analysis is tedious and somewhat uncertain. A freshly prepared article crystallizes at about 70°. At 90° to 95° it is entirely liquefied. Unless contaminated by combination with organic matter, it is nearly colorless.

PHYSIOLOGICAL EFFECTS. — Carbolic acid is a prompt, active escharotic. Its action depends on its affinity for albumen, with which it forms a white compound. Its action is much like that of creosote, but is more prompt, and, therefore, less painful when applied to soft tissues.

USES.—It may be used in any case when creosote is indicated. It is better than creosote as an application to indolent or apthous ulcers.

### Chloride of Zinc.—ZnCl.

Preparation.—It may be prepared by dissolving zinc in hydrochloric acid, evaporating to dryness and fusing in a Florence flask. Prepared thus, it contains traces of oxyd of zinc. To remove this, a little nitric acid may be added before evaporating to dryness. The dry mass may then be dissolved in water, and prepared chalk added as long as effervescence takes place, when the mass is to be again evaporated to dryness. But, as the article is kept at all drugstores, we will seldom have occasion to prepare it.

Properties.—It is a whitish, semi-transparent mass, having the consistence of hard butter—and is hence called "butter of zinc." It deliquesces in the air, and is soluble in water, alcohol and ether. It fuses a little above 212,° and sublimes at a red heat. It combines with albumen and gelatin, forming compounds which are but slightly soluble in an excess of either agent.

Physiological Effects.—Chloride of zinc is an escharotic, by virtue of its affinity for albumen, gelatin and water. It destroys the vitality of parts to which it is applied by abstracting these compounds. On account of the same affinities, it acts as an antiseptic; and on account of others less obvious, it is also a disinfectant.

Uses.—The dentist has no use for chloride of zinc, except to destroy the sensibility of dentine. Any other purpose for which it might be used, will be better subserved by some other agent. When applied to dentine in substance, or in the solid state, it acts but little, if at all, on the earthy materials; but, by decomposing the animal matter, it destroys the vitality of the dentine, just to the extent of the chemical combinations which take place. By destroying the vitality of the surface, a shock is given to the subjacent parts, which will be followed by re-action, which is increased vitality, resulting, of course, in increased sensibility. To gain anything, therefore, by this medicine, we must either operate before this re-action begins, or wait till it has subsided. This re-action will be more prompt in some constitutions than in others—generally more rapid in the young than in the old. Just how soon it will come up and how soon subside, can not be determined by rule. Doubtless many fail to get satisfactory results, by a failure to recognize this principle, which is common to the action of all escharotics. Applying the chloride to a sensitive tooth to-day, for the purpose of operating to-morrow, is ordinarily but adding fuel to the fire. It is better to operate, in most cases, within twenty or thirty minutes, or to await the lapse of several days.

### TERCHLORIDE OF GOLD, $AuCl_3$.

PREPARATION.—Dissolve gold in *aqua regia*, composed of three parts, by measure, of hydrochloric acid and one of nitric acid. Gentle heat will hasten the solution. The acids should be chemically pure. Evaporate the solution to dryness, and the result will be ruby-red, prismatic crystals of the terchloride of gold.

PROPERTIES. — Soluble in water, alcohol and ether; is highly deliquescent, has a disagreeable, styptic taste, reddens litmus, gives a purple stain to the cuticle, which is removed by a solution of cyanide of potassium. Its elements are held together by a feeble affinity, hence it is readily decomposed by many metallic and non-metallic elements, as well as by saline and organic compounds.

PHYSIOLOGICAL EFFECTS.—These are, in many respects, similar to those of corrosive sublimate, or chloride of mercury. It combines with albuminous and gelatinous substances; and its affinities for these are so strong that it takes them from living tissues, hence it is an escharotic. In contact with organic matter, a part of it is decomposed, and chlorine is liberated; hence it acts as a disinfectant.

USES.—The dentist has no call for its internal administration. To destroy the sensibility of dentine, he will find it a valuable application. And for this purpose, the ethereal solution has probably some advantages over the aqueous or the alcoholic. It is readily prepared as follows: Dissolve the crystals, as above prepared, in pure water, fill a test tube, or long phial, half full of the solution, then add an equal quantity of sulphuric ether and agitate the mixture. Let it then rest a few minutes, the ethereal solution will then rise to the surface, and may be poured off into another phial, and securely corked. It should be kept, as much as practicable, from the action of light and air.

Applied to dentine, on pledgets of cotton, it acts like chloride of zinc, but more promptly, and with less pain. Chlorine is more abundantly liberated during its action than from chloride of zinc, hence it is a better disinfectant than the latter.

